# Supplementary material for: Culture-Dependent and Metabarcoding Characterization of the Sugar Beet (Beta vulgaris L.) Microbiome for High-Yield Isolation of Bacteria with Plant Growth-Promoting Traits
Source: Microorganisms. 2023 Jun 9;11(6):1538. doi: 10.3390/microorganisms11061538 (PMC10302512; doi:10.3390/microorganisms11061538)
Supplement: Supplementary file 1 [file microorganisms-11-01538-s001.zip › microorganisms-2392846-supplementary.pdf]

microorganisms

Article

Culture-Dependent and Metabarcoding Characterization of the Sugar Beet (*Beta vulgaris* L.) Microbiome for High-Yield Isolation of Bacteria with Plant Growth-Promoting Traits

Tamara Krstić Tomić [1,†], Iva Atanasković [1,2,†], Ivan Nikolić [1,2], Nataša Joković [3], Tatjana Stević [4], Slaviša Stanković [1,2], Tanja Berić [1,2] and Jelena Lozo [1,2,*]

[1] University of Belgrade, Faculty of Biology, 11000 Belgrade, Serbia; m3004_2020@stud.bio.bg.ac.rs (T.K.T.); iva.atanaskovic@bio.bg.ac.rs (I.A.); ivan.nikolic@bio.bg.ac.rs (I.N.); slavisas@bio.bg.ac.rs (S.S.); tanjab@bio.bg.ac.rs (T.B.)
[2] University of Belgrade, Faculty of Biology, Centre for Biological Control and Plant, Growth Promotion, 11000 Belgrade, Serbia
[3] Faculty of Sciences and Mathematics, University of Niš, 18000 Niš, Serbia; natasa.jokovic@pmf.edu.rs
[4] Institute for Medicinal Plants Research "Dr. Josif Pančić", 11000 Belgrade, Serbia; tanjasomosa@gmail.com
* Correspondence: jlozo@bio.bg.ac.rs.
† These authors contributed equally to this work.

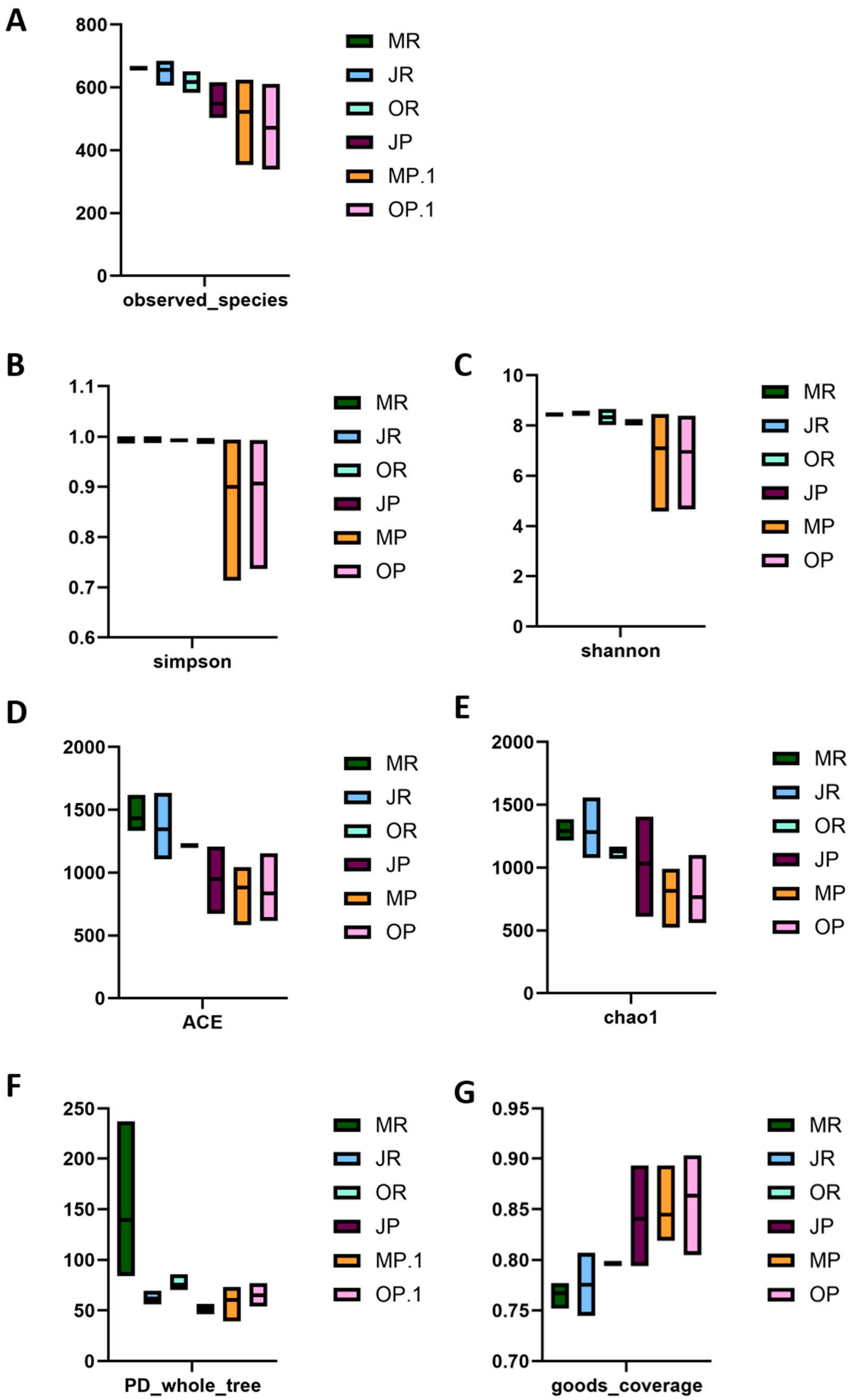
A
800
600
400
200
0
observed_species
MR
JR
OR
JP
MP.1
OP.1
B
1.1
1.0
0.9
0.8
0.7
0.6
simpson
MR
JR
OR
JP
MP
OP
C
10
8
6
4
2
0
shannon
MR
JR
OR
JP
MP
OP
D
2000
1500
1000
500
0
ACE
MR
JR
OR
JP
MP
OP
E
2000
1500
1000
500
0
chao1
MR
JR
OR
JP
MP
OP
F
250
200
150
100
50
0
PD_whole_tree
MR
JR
OR
JP
MP.1
OP.1
G
0.95
0.90
0.85
0.80
0.75
0.70
goods_coverage
MR
JR
OR
JP
MP
OP

Figure S1. Parameters of alpha-diversity for rhizosphere (R) and phyllosphere (P) samples collected in May (M), July (J), or October (O).

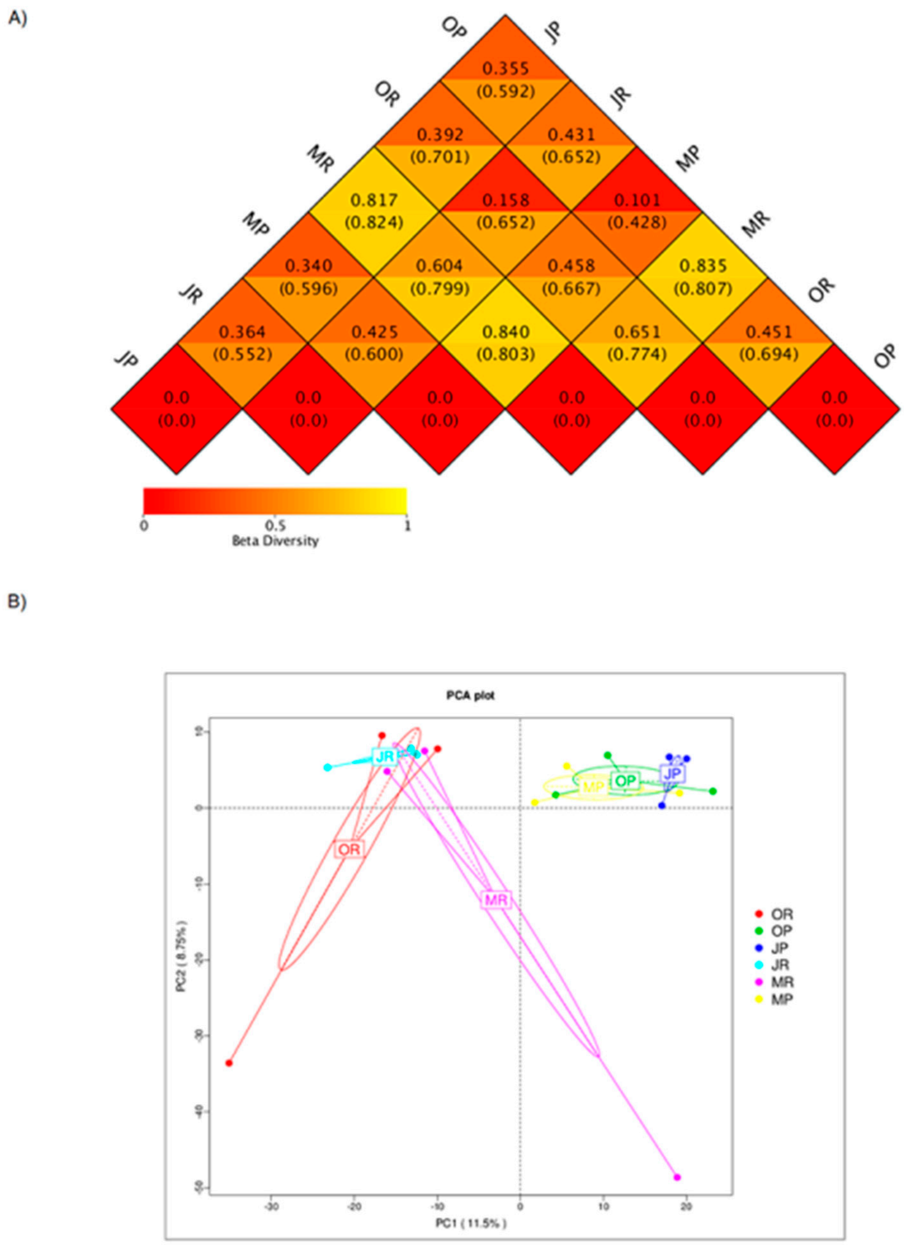

Figure S2. Beta diversity analysis of the sugar beet microbiome. (A) Heatmap shows weighted UniFrac and unweighted UniFrac distances. Grids represent the pairwise dissimilarity coefficient between samples (weighted UniFrac distance is shown at the top and unweighted UniFrac distance is shown in reverse); (B) PCA plot based on weighted UniFrac distances. In PCA plots, the x-axis represents the first principal component, the y-axis represents the second principal component, and the percentages represent the contribution of the principal component to the sample difference; phyllosphere (P), rhizosphere (R) samples collected in May (M), July (J), or October (O).

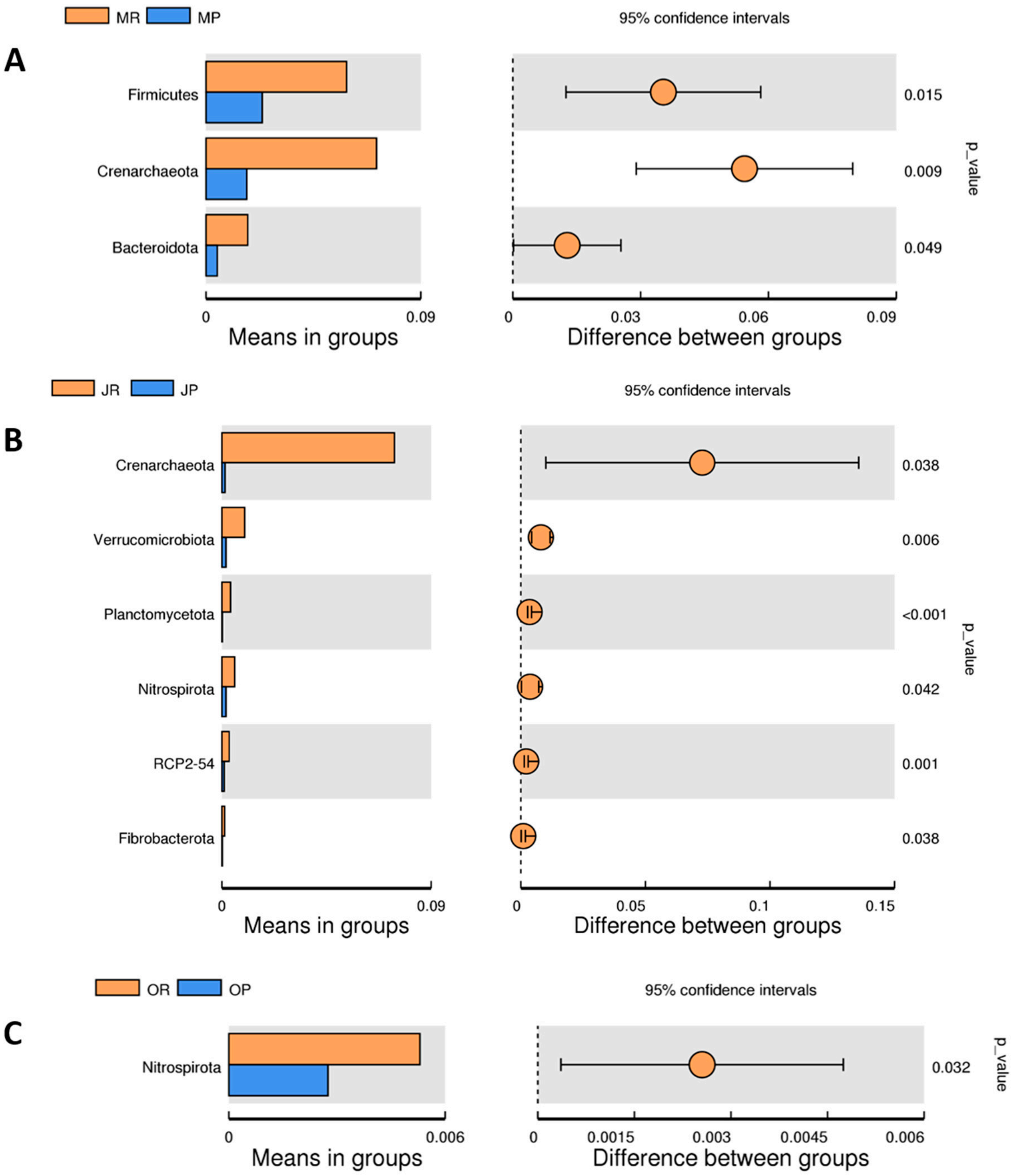

Figure S3. Comparison of phylum abundance of the rhizosphere (R) and phyllosphere (P) samples obtained in May (A), July (B), and October (C). The left panel shows the frequency of OTUs that show a significant difference (p-value<0.05) between groups, with each bar representing the mean frequency. The right panel represents the confidential interval of group variation. The left part of each circle represents the lower limit of the 95% confidential interval, while the right part represents the upper limit. The center of the circle represents the difference in the mean. The color of the circle corresponds to the group whose mean is higher.

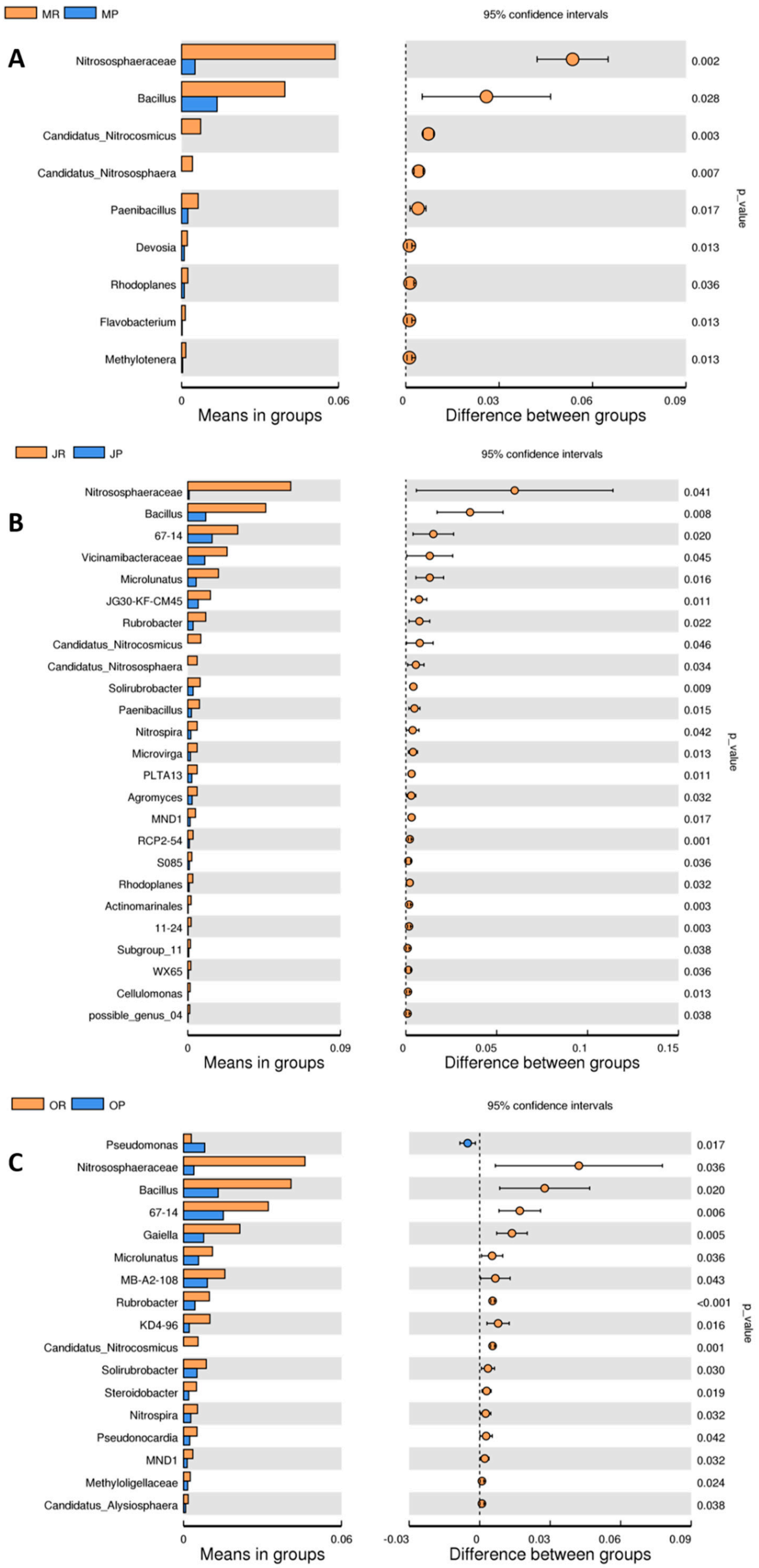
MR MP
95% confidence intervals
A
Nitrososphaeraceae 0.002
Bacillus 0.028
Candidatus_Nitrocosmicus 0.003
Candidatus_Nitrososphaera 0.007
Paenibacillus 0.017
Devosia 0.013
Rhodoplanes 0.036
Flavobacterium 0.013
Methylotenera 0.013
p_value
Means in groups
Difference between groups
JR JP
95% confidence intervals
B
Nitrososphaeraceae 0.041
Bacillus 0.008
67-14 0.020
Vicinamibacteraceae 0.045
Microlunatus 0.016
JG30-KF-CM45 0.011
Rubrobacter 0.022
Candidatus_Nitrocosmicus 0.046
Candidatus_Nitrososphaera 0.034
Solirubrobacter 0.009
Paenibacillus 0.015
Nitrospira 0.042
Microvirga 0.013
PLTA13 0.011
Agromyces 0.032
MND1 0.017
RCP2-54 0.001
S085 0.036
Rhodoplanes 0.032
Actinomarinales 0.003
11-24 0.003
Subgroup_11 0.038
WX65 0.036
Cellulomonas 0.013
possible_genus_04 0.038
p_value
Means in groups
Difference between groups
OR OP
95% confidence intervals
C
Pseudomonas 0.017
Nitrososphaeraceae 0.036
Bacillus 0.020
67-14 0.006
Gaiella 0.005
Microlunatus 0.036
MB-A2-108 0.043
Rubrobacter <0.001
KD4-96 0.016
Candidatus_Nitrocosmicus 0.001
Solirubrobacter 0.030
Steroidobacter 0.019
Nitrospira 0.032
Pseudonocardia 0.042
MND1 0.032
Methyloligellaceae 0.024
Candidatus_Alysiosphaera 0.038
p_value
Means in groups
Difference between groups

Figure S4. Comparison of genus abundance of the rhizosphere (R) and phyllosphere (P) samples obtained in May (A), July (B), and October (C). The left panel shows the frequency of OTUs that show a significant difference (p-value<0.05) between groups, with each bar representing the mean frequency. The right panel represents the confidential interval of group variation. The left part of each circle represents the lower limit of the 95% confidential interval, while the right part represents the upper limit. The center of the circle represents the difference in the mean. The color of the circle corresponds to the group whose mean is higher.

Table S1. QC statistics for 16S rRNA gene metabarcoding. Amplicon was sequenced on Illumina paired-end platform to generate 250 bp paired-end raw reads (Raw PE), and then merged and pretreated to obtain Clean Tags. The chimeric sequences in Clean Tags were detected and removed to obtain the Effective Tags which can be used for subsequent analysis. The summarizations obtained in each step of data processing are shown. Raw PE represents the original PE reads after sequencing. Raw Tags represent tags merged from PE reads. Clean Tags represent tags after filtering. Effective Tags represent tags after filtering chimera and can be finally used for subsequent analysis. The base is the number of bases of the Effective Tags. AvgLen represents the average length of Effective Tags. Q20 and Q30 are the percentages of bases whose quality value in Effective Tags is greater than 20 (sequencing error rate is less than 1%) and 30 (sequencing error rate is less than 0.1%). GC (%) represents GC content in Effective Tags. Effective (%) represents the percentage of Effective Tags in Raw PE.

Sample Name	Raw PE(#)	Combined(#)	Qualified(#)	Nochime(#)	Base(nt)	AvgLen(nt)	Q20	Q30	GC%	Effective%
JP.1	186,213	142,710	139,260	118,128	########	407	97.6	92.76	56.29	63.44
JP.2	178,785	143,451	141,271	137,894	########	406	97.6	92.55	56.17	77.13
JP.3	172,684	141,620	139,417	135,432	########	406	97.61	92.7	56.22	78.43
JR.1	189,900	155,091	151,890	108,583	########	409	97.58	92.7	56.57	57.18

Copyright: © 2023 by the authors. Licensee MDPI, Basel, Switzerland. This article is an open access article distributed under the terms and conditions of the Creative Commons Attribution (CC BY) license (https://creativecommons.org/licenses/by/4.0/).

JR.2	178,701	140,594	136,624	85,896	########	410	97.48	92.33	56.89	48.07
JR.3	173,695	142,964	139,377	69,264	########	412	97.53	92.29	57.37	39.88
MP.1	185,511	153,766	151,333	145,780	########	406	97.74	93.06	56.24	78.58
MP.2	189,109	146,718	144,254	140,013	########	406	97.61	92.72	56.19	74.04
MP.3	184,624	144,267	141,919	135,966	########	406	97.63	92.78	56.26	73.64
MR.1	180,860	143,706	139,961	84,551	########	410	97.47	92.36	56.83	46.75
MR.2	309,799	220,444	205,041	164,312	########	404	97.13	91.88	55.79	53.04
MR.3	188,469	152,093	148,157	107,763	########	408	97.7	92.87	56.56	57.18
OP.1	173,908	135,403	133,236	128,175	########	406	97.56	92.62	56.23	73.7
OP.2	187,768	145,717	143,339	138,264	########	406	97.51	92.46	56.14	73.64
OP.3	187,936	141,723	139,363	133,942	########	406	97.5	92.47	56.29	71.27
OR.1	182,797	146,668	142,702	79,538	########	411	97.38	92.13	57.18	43.51
OR.2	171,543	147,907	142,320	83,585	########	413	98.95	96.33	56.27	48.73
OR.3	181,048	152,511	148,922	75,251	########	411	97.58	92.54	57.17	41.56

Table S2. Characteristics of isolates obtained from the rhizosphere and phyllosphere of sugar beet in different seasons. Each isolate has a unique designation, with M denoting isolates from May, J from July, and O from October. R and Rh stand for isolates from the rhizosphere and L for isolates from the phyllosphere. The isolates are paired with the culture media on which they were detected. Individual traits are grouped in columns and the presence of a trait is marked with +.

ISOLATE	MEDIUM	EXOPOLYSACCNARIDE PRODUCTION	SIDEROPHORE PRODUCTION	HCN PRODUCTION	PHOSPNATE SOLUBILIZATION	Ps CFBR2437	Ps P16	Ps P21	*F. oxysporum*	*Rh. Solani*	SEED GERMINATION	NUMBER OF TRAITS	SPECIES
JL 1	LEa											0	
JL 10	LEph	+			+				+			3	*Pantoea agglomerans, Enterobacter ludwigii*
JL 11	LEph	+			+	+	+	+			+	6	*Paenibacillus massiliensis*
JL 12.1	LEph								+			1	
JL 13	LEph	+	+		+				+			4	
JL 14.2	LEph		+		+	+	+	+	+	+		7	*Acinetobacter courvalinii*
JL 15	LEph					+	+	+	+			4	*Stenotrophomonas maltophilia*
JL 2	LEa						+	+	+			3	*Stenotrophomonas maltophilia*
JL 20	LEph								+	+	+	3	*Paenibacillus massiliensis*
JL 22	LEph								+	+		2	
JL 23	NA					+	+	+	+	+		5	*Brucella grignonensis*
JL 26	NA					+	+	+				3	
JL 3	LEa	+			+	+	+	+				5	*Brucella pituitosa*
JL 30	NA			+	+	+	+	+	+	+		7	*Stenotrophomonas maltophilia*
JL 35	NA					+	+	+	+			4	
JL 37	NA					+	+	+		+		4	*Stenotrophomonas maltophilia*
JL 39	PS								+	+		2	
JL 4	LEa				+	+	+	+				4	
JL 41	PS				+	+	+	+	+	+		6	*Stenotrophomonas maltophilia*
JL 42	PS				+							1	
JL 44	PS				+				+	+		3	
JL 47	LEa					+	+	+	+	+		5	*Stenotrophomonas maltophilia*

JL 48	LEa								+	+		2	
JL 49	LEa								+			1	
JL 49.2	LEa								+			1	
JL 5	LEa	+			+	+	+	+	+	+		7	*Stenotrophomonas maltophilia*
JL 52	LEa				+				+			2	
JL 53	LEa	+			+				+			3	
JL 54	LEa								+			1	*Streptomyces venezuelae, S. zaomyceticus, S. lateritius*
JL 55	LEa					+	+	+	+			4	*Stenotrophomonas maltophilia*
JL 56	LEph				+	+	+	+	+			5	*Stenotrophomonas maltophilia*
JL 6	LEa				+				+			2	
JL 60	LEph					+	+	+	+			4	
JL 65	LEph	+								+		2	*Bacillus zanthoxyli, Priestia aryabNAttai*
JL 66	NA		+		+	+	+	+	+	+		7	
JL 67	NA					+	+	+	+	+		5	*Stenotrophomonas maltophilia*
JL 71	NA					+	+	+	+	+		5	*Stenotrophomonas maltophilia*
JL 72	NA				+				+	+		3	
JL 74	NA				+	+	+	+		+		5	*Brucella pseudogrignonensis*
JL 76.2	PS	+								+		2	
JL 79	PS	+	+		+		+	+	+			6	
JL 8	LEa		+									1	
JL 80	PS				+				+	+		3	*Pantoea agglomerans, Enterobacter ludwigii*
JL 9	LEa				+	+	+	+	+			5	*Stenotrophomonas maltophilia*
ML 10	NA				+	+	+	+	+	+		6	*Stenotrophomonas tumulicola*
ML 11	NA										+	1	*Peribacillus frigoritoLEarans*
ML 15	PS					+	+	+	+	+	+	6	
ML 15	PS					+	+	+	+	+	+	6	*Bacillus thuringiensis*
ML 156	LEa											0	
ML 158	LEa	+									+	2	*Curtobacterium herbarum*
ML 159	LEa	+	+		+				+	+	+	6	*Pseudomonas lurida*
ML 16	PS											0	
ML 162	NA										+	1	*Bacillus sp.*

ML 164	PS											0	
ML 166	LEa											0	
ML 168	LEa	+	+									2	
ML 169.2	LEa	+	+							+		3	
ML 17	PS										+	1	*Metabacillus dongyingensis*
ML 170	LEa	+	+			+	+	+	+	+		7	*Pseudomonas grimontii*
ML 171	NA											0	
ML 172	NA	+	+									2	
ML 173	NA											0	*Micrococcus yunnanensis*
ML 18	PS										+	1	*Metabacillus dongyingensis*
ML 19	NA	+	+									2	
ML 2	LEph	+	+								+	3	*Pseudomonas lutea*
ML 20	NA		+			+	+					3	
ML 21	NA											0	*Curtobacterium allii*
ML 25	NA									+		1	*Arthrobacter ginsengisoli*
ML 254	LEa		+									1	
ML 255	NA											0	
ML 257	NA											0	
ML 26	NA											0	
ML 27	NA											0	
ML 28	PS											0	*Pseudomonas paracarnis*
ML 3	LEph											0	
ML 30	PS											0	
ML 31	PS					+	+		+			3	
ML 5	NA				+				+	+		3	
ML 8	NA		+									1	
OL 104	LEph								+		+	2	*Pseudomonas grimontii, Arthrobacter subterraneus, A. parietis, A. tumbae*
OL 108	NA											0	
OL 109	NA				+					+		2	*Pantoea agglomerans*
OL 112	NA								+			1	*Pseudarthrobacter psychrotoLEarans*

OL 113.1	NA											0	
OL 114	NA											0	
OL 119.1	NA											0	
OL 120	NA								+			1	*Oceanobacilluas chungangensis*
OL 254	NA		+						+			2	*Staphylococcus pseudoxylosus, S. saprophyticus*
OL 257	NA		+						+			2	
OL 101	LEa	+			+				+	+		4	*Erwinia persicina*
OL 102	LEa								+			1	
OL 105	LEph								+			1	
OL 113.2	NA								+			1	*Acinetobacter lwoffii*
OL 118.2	NA		+		+								*Arthrobacter subterraneus*
OL 119.2	NA								+			1	*Promicromonospora xylanilytica*
OL 122	PS								+	+		2	*Pectobacterium brasiliense*
OL 123	PS								+			1	
OL 126	LEa	+			+							2	*Pantoea agglomerans, Curtobacterium plantarum*
OL 127	LEa	+			+							2	*Erwinia persicina*
OL 128	LEa											0	
OL 129	LEa	+	+			+	+	+				5	
OL 131	LEa				+				+			2	
OL 133	LEa		+		+				+			3	*Enterobacter quasihormaechei, E. hormaechei*
OL 138	LEph	+	+		+				+			4	
OL 139	LEph	+							+			2	
OL 140	LEph		+						+			2	*Exiguobacterium artemiae*
OL 141	LEph					+	+	+	+			4	*Pseudomonas grimontii*
OL 146	NA	+	+		+				+			4	
OL 148	NA	+	+		+				+			4	*Pantoea agglomerans*
OL 149	NA	+	+		+							3	*Erwinia persicina*

OL 150	NA										+	1	*Curtobacterium oceanosedimentum*
OL 151	NA											0	
OL 152	NA								+			1	*Cytobacillus oceanisediminis*
OL 154	NA	+	+									2	
OL 155	NA	+	+		+				+			4	
OL 157	NA											0	
OL 158	NA								+			1	*Pseudomonas arenae*
OL 159	NA	+										1	
OL 161	PS	+			+				+			3	
OL 162	PS											0	*Pseudomonas baltica, P. coLEaopterorum*
OL 164	PS	+			+				+	+		4	*Pantoea agglomerans*
OL 166	PS											0	
OL 167	PS	+			+				+	+		4	*Pantoea agglomerans*
OL 168	PS	+			+				+	+		4	
OL 253	LEph		+									1	
OL 260	NA								+		+	2	*Micrococcus luteus, M. aloeverae; Enterobacter ludwigii, E. cloacae*
OL 262	PS	+				+	+	+	+	+		6	
OL 293	PS											0	
